# Deletion analysis of BMI1 oncoprotein identifies its negative regulatory domain

**DOI:** 10.1186/1476-4598-9-158

**Published:** 2010-06-22

**Authors:** Ajay K Yadav, Anagh A Sahasrabuddhe, Manjari Dimri, Prashant V Bommi, Rachana Sainger, Goberdhan P Dimri

**Affiliations:** 1 Department of Medicine, NorthShore University HealthSystem Research Institute, Evanston, IL 60201, USA; 2 Dr. B.R Ambedkar Centre for Biomedical Research, University of Delhi Delhi-110007, India; 3 Department of Surgery, Cardiovascular, University of Pennsylvania, PA 19036, USA

## Abstract

**Background:**

The polycomb group (PcG) protein BMI1 is an important regulator of development. Additionally, aberrant expression of BMI1 has been linked to cancer stem cell phenotype and oncogenesis. In particular, its overexpression has been found in several human malignancies including breast cancer. Despite its established role in stem cell maintenance, cancer and development, at present not much is known about the functional domains of BMI1 oncoprotein. In the present study, we carried out a deletion analysis of BMI1 to identify its negative regulatory domain.

**Results:**

We report that deletion of the C-terminal domain of BMI1, which is rich in proline-serine (PS) residues and previously described as PEST-like domain, increased the stability of BMI1, and promoted its pro-oncogenic activities in human mammary epithelial cells (HMECs). Specifically, overexpression of a PS region deleted mutant of BMI1 increased proliferation of HMECs and promoted an epithelial-mesenchymal transition (EMT) phenotype in the HMECs. Furthermore, when compared to the wild type BMI1, exogenous expression of the mutant BMI1 led to a significant downregulation of p16INK4a and an efficient bypass of cellular senescence in human diploid fibroblasts.

**Conclusions:**

In summary, our data suggest that the PS domain of BMI1 is involved in its stability and that it negatively regulates function of BMI1 oncoprotein. Our results also suggest that the PS domain of BMI1 could be targeted for the treatment of proliferative disorders such as cancer and aging.

## Background

Polycomb Group (PcG) proteins originally discovered in Drosophila are evolutionarily conserved epigenetic regulators of development [1-3]. These proteins regulate proliferation and differentiation of cells via epigenetic silencing of important growth regulatory genes [3,4]. The first mammalian PcG gene *BMI1 *(B lymphoma Mo-MLV insertion region 1) was identified as a c-myc cooperating oncogene using an Eμ-myc transgenic mouse model [5,6]. There is increasing evidence that the deregulated expression of BMI1 contributes to cancer development. It is overexpressed in a number of cancers, such as mantle cell lymphoma [7], B-cell non-Hodgkin's lymphoma [8], myeloid leukemia [9], non-small cell lung cancer [10], colorectal cancer [11], breast and prostate cancers [12,13], and head and neck cancers [14,15]. In addition to its role in cancer, BMI1 is also known to be required for self-renewal of neural, hematopoietic, intestinal and mammary stem cells [16-21]. Consistent with its role in stem cell self-renewal, BMI1 expression is thought to promote stem-ness in tumor cells [12,22], and BMI1 is considered an important marker of breast cancer stem cells [23]. Recent mouse xenograft studies using BMI1 and Ras co-overexpressing human mammary epithelial cells (HMECs) also support oncogenic roles for BMI1 in breast cancer development and metastasis of breast cancer cells [24,25].

PcG proteins assemble into polycomb repressive complexes (PRCs), which possess histone posttranslational modification (PTM) activities and act in a sequential fashion to mediate gene silencing [3]. Biochemically, BMI1 is a core component of PRC1, which ubiquitinates histone 2A at lysine 119 residue [26], and acts downstream of PRC2, which trimethylates lysine 27 residue of histone 3 [27,28]. Although BMI1 is a prominent component of PRC1, its exact role in PRC1 is unclear. BMI1 by itself does not appear to have an E3 ubiquitin ligase activity [29], instead, the E3 ubiquitin ligase activity of PRC1 strictly depends on Ring1B (RING2) protein. However, it has been shown that Ring1B-mediated E3 ubiquitin ligase activity of PRC1 complex is enhanced by BMI1 [29-31].

Structurally, human BMI1 is comprised of 326 amino acids [32]. The primary structure of BMI1 in mice revealed the presence of a RING finger (RF) domain at the N-terminus, a potential HTH (helix turn helix) domain in the middle and a PEST (proline (P), glutamic acid (E), serine (S) and threonine (T) rich) -like domain at the C-terminus [5,6]. These domains of BMI1 are highly conserved across mammalian species including human. The BMI1 also contains two putative nuclear localization signals (NLS), NLS1 (KRRR, amino acid residues 92-95) and NLS2 (KRMK, amino acid residues 232-235). Of these two, only NLS2 appears to be functional in targeting BMI1 to the nucleus in mouse and human cells [33,34]. We have previously carried out functional analysis of BMI1 and shown that the RING finger and HTH domains of BMI1 are required for downregulation of p16INK4a tumor suppressor and bypass of senescence in human diploid fibroblasts (HDFs) [35]. We also showed that both of these domains are required for immortalization of normal HMECs [34].

PEST-like domains rich in proline (P), glutamic acid (E), serine (S) and threonine (T) residues have been described in the literature [36]. Although the actual contribution of such domains to protein turn-over is not clear and such sequences may not mediate proteolysis per se, the PEST-like domains are present in many proteins that undergo rapid turn-over such as cyclins, NF-κβ, c-Myc, c-Fos, ODC, ABCA1 etc [36-44]. Since a PEST-like domain, which is rich in proline (P) and serine (S) residues is present in BMI1 [5,6], and at present very little is known about its functional role, we carried out a structural-functional analysis of BMI1 to define the role of this domain in BMI1. As the PEST-like domain in BMI1 is not rich in E- and T- residues, here in, we refer this domain as the PS domain. Our data indicate that the PS domain of BMI1 may indeed regulate its proteolysis and that it may function as a negative regulatory domain of BMI1.

## Results

### Deletion of the PS domain results in increased half life of BMI1

**Figure 1:**
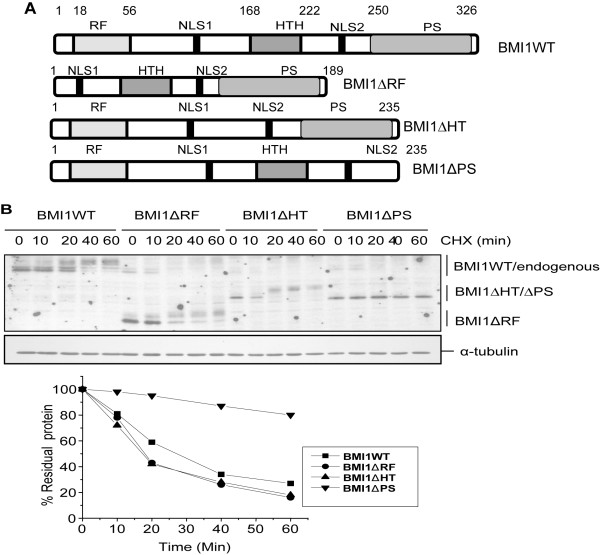
**Deletion of the PS domain of BMI1 results in increased half life of BMI1**. (A). Schematic representation of wild type BMI1 and its deletion mutants. The domain structure of the wild type BMI1, and the ∆HT and ∆RF mutants has been described previously [34,35]. The ∆PS mutant of BMI1 encodes a protein, which lacks amino acids 236- 326. Its cDNA was generated by PCR and cloned in the pBabe- puro retroviral vector. (B). The half life of wild type and mutant BMI1 in MCF10A cells expressing the respective protein was determined by blocking synthesis of new proteins using 100 μg/ml CHX for different time points (0-60 min) and the rate of BMI1 degradation was determined by western blot analysis of remaining BMI1 protein after each time point and normalizing it to ß-actin using densitometry measurements as described in the Materials and Methods section. In case of the wild type, and the ΔRF and the ΔHT mutants, only the lower most band of BMI1, which showed a time-dependent decrease was quantified to determine the half life. The densitometric analysis was performed using Image J software (NIH, Bethesda, MD), and the graph was generated by plotting % residual protein vs time of CHX treatment.

We have previously reported that the RING finger and HTH domains of BMI1 are required for its pro-proliferative activities and to enable bypass of senescence in HDFs and HMECs [34,35]. To further understand the role of the various domains of BMI1, we generated a PS deletion mutant which we termed as ΔPS mutant (Fig. 1A), and used it as a tool to study the biochemical and posttranslational regulation of BMI1. MCF10A cells stably expressing wild type (WT), and ΔRF, ΔHT and ΔPS mutants of BMI1 were selected in puromycin as described in the Materials and Methods section. The drug-selected cells were further passaged in culture and the half lives of wild type and mutant BMI1 proteins were determined using cyclohexamide (CHX) treatment at different time points. Our results indicated that the wild type BMI1 has a half life of ~25 minutes and that the ΔRF, and ΔHT mutants degraded faster than the wild type BMI1 with a half life of 15-20 minutes (Fig. 1B). On the other hand, ΔPS mutant of BMI1 was relatively very stable and did not undergo any significant proteolysis up to 60 minutes (Fig. 1B).

**Figure 2:**
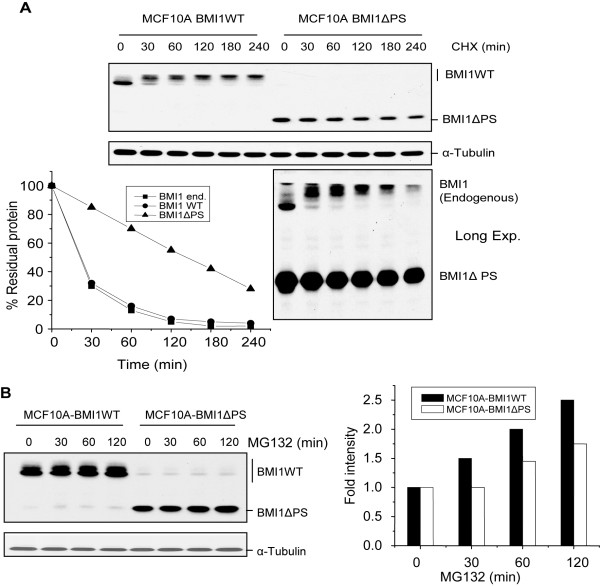
**The ΔPS mutant of BMI1 is more stable than the wild type BMI1**. (A).The half life of the ΔPS mutant of BMI1 and the wild type BMI1 was determined using CHX treatment (0-240 min) of MCF10A-BMI1WT and MCF10A-BMI1ΔPS cells as described above. (B). MCF10A-BMI1WT and MCF10A-BMI1ΔPS cells were treated with MG132 for different times points (as indicated) to determine its effect on the wild type and the ΔPS mutant of BMI1. Cells were harvested after each time point and analyzed for accumulation of the wild type or mutant BMI1 protein by western blot analysis. The accumulated proteins were quantified by densitometric analysis of signal present in respective lanes and by normalizing it to the individual α-tubulin signal. The densitometric analysis was done as described in Fig. 1B.

To determine the half life of the ΔPS mutant of BMI1, MCF10A-BMI1WT and MCF10A-BMI1ΔPS cells were further treated with CHX for longer periods (0-240 min) and the half lives of the wild type BMI1 and ΔPS mutant were determined as described above. Our results indicated that the half life of ΔPS mutant of BMI1 is about 120 min (Fig. 2A). We also determined the rate of proteolysis of endogenous BMI1 in MCF10A-BMI1ΔPS cells. Our analysis of the half life of BMI1 proteins indicated that, similar to the overexpressed wild type BMI1, the endogenous BMI1 also has a half life of 25-30 min in MCF10A cells (Fig. 2A lower panel).

During our analysis of BMI1 half life, we noticed that the wild type but not the ΔPS mutant of BMI1 is detected as multiple bands of different mobility, and that the intensity of the slow mobility bands increases with CHX treatment. To determine whether the slow mobility bands represent phosphorylated forms of BMI1, we treated total cell extract of MCF10A-BMI1 cells with calf intestinal alkaline phosphatase (CIP) and performed an immunoblot analysis (IB) of BMI1. Our results indicated that the slower mobility bands of BMI1 likely do represent phosphorylated forms of BMI1, which disappear upon CIP treatment (Additional file 1, Fig. S1A).

To further confirm the relatively stable nature of the ΔPS mutant of BMI1, we treated MCF10A-BMI1WT and MCF10A-BMI1ΔPS cells with a proteosome inhibitor MG132 at different time points. We reasoned that if the ΔPS mutant is more stable than the wild type BMI1 and is not degraded as fast as the wild type, it will not accumulate upon MG132 treatment, which is a commonly used to inhibit 26S proteosome. Indeed, our results indicated that the wild type BMI1 is accumulated within 30 min upon MG132-treatment, while the ΔPS mutant of BMI1 had not accumulated after 30 min of treatment (Fig. 2B). Our data also indicated that longer treatment of MG132 results in accumulation of ΔPS mutant of BMI1; however, compared to the wild type, the relative accumulation of ΔPS mutant of BMI1 was much less pronounced at all the time points we analyzed (Fig. 2B).

To determine whether BMI1 is non-specifically degraded by proteases, we treated cells with two commonly used protease inhibitors; phenylmethylsulfonyl fluoride (PMSF) and aprotinin. We also used lactacystin, another specific inhibitor of 26S-proteosome. After treatment of the cells with PMSF, aprotinin and lactacystin, BMI1 was detected by IB analysis. Our results indicated that BMI1 is accumulated only in lactacystin-treated cells (Additional file 1, Fig. S1B). Thus, BMI1 is specifically degraded by a 26S proteosome-mediated protein degradation pathway. Finally, we also determined the half life of wild type BMI1 and ΔPS mutants in HDFs. The results indicate that similar to what we saw in MCF10A cells, the wild type BMI1 has a half life of ~25 min in MRC5 and IMR90 strains of HDFs, while the ΔPS mutant of BMI1 is very stable in these cells (Additional file 1, Fig. S2 and Fig. S3). Collectively, our data suggest that deletion of the PS domain of BMI1 renders it much more stable compared to the overexpressed or endogenous wild type BMI1.

### Deletion of the PS domain of BMI1 promotes proliferation of cells

**Figure 3:**
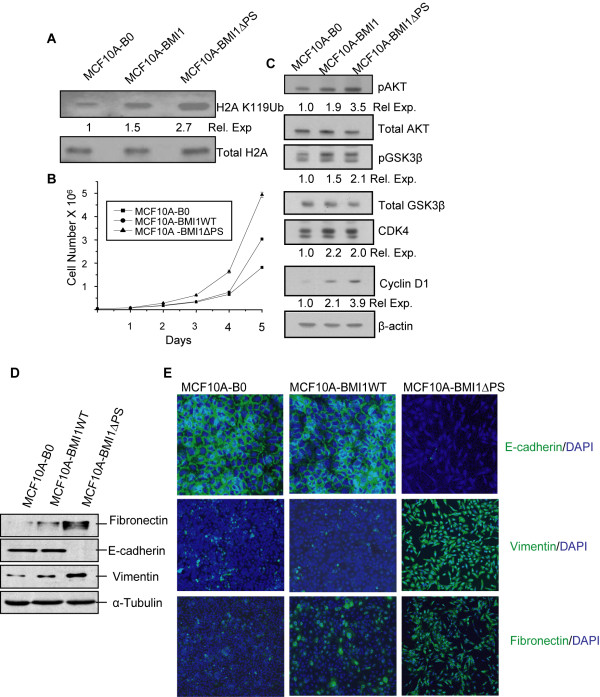
**Deletion of the PS domain of BMI1 augments its pro-proliferative activity and promotes EMT in MCF10A cells**. (A). Wild type BMI1 and ΔPS mutant of BMI1 upregulates H2A K119Ub activity of PRC1. The relative expression of H2A K119Ub in MCF10A control, MCF10A-BMI1WT and MCF10A-BMI1ΔPS cells was determined by western blot analysis and densitometry as described in the Materials and Methods section and Fig. 1B. (B). MCF10A-derived cells expressing wild type and mutant BMI1 were plated in P100 plastic dishes (5 × 10^5^/plate). Cells were harvested at the indicated day (days 1-5) and counted using a hemocytometer. Proliferation curves were generated by plotting the number of cells against number of days. (C). Increase in AKT activity (phospho-AKT and phospho-GSK3β), and the expression of AKT/GSK3β targets cyclin D1 and CDK4 was determined by western blot analysis using antibody specific to each protein as described in the Materials and Methods section. β-actin was used as a loading control. The relative expression of phospho-AKT (normalized to total AKT), Phospho-GSK3β (normalized to total GSK3β), CDK4 (normalized to β-actin) and cyclin D1 (normalized to β-actin) was quantified by densitometry as described in Fig. 1B. (D). MCF10A-derived cells expressing wild type BMI1 or the ∆PS mutant were analyzed for the expression of E-cadherin, fibronectin and vimentin using western blot analysis. α-tubulin was used as a loading control. (E). The expression of E-cadherin, vimentin and fibronectin in control, and wild type BMI1- and ∆PS mutant-overexpressing cells was determined by immunostaining as described in the Materials and Methods section. After immunostaining, cells were photographed (10X) using a Nikon Eclipse 80i confocal microscope.

To determine the possible consequences of deleting the PS region of BMI1 on PRC1 activity, we examined the levels of H2A K119Ub in MCF10A-B0 control, MCF10A-BMI1WT and MCF10A-BMI1∆PS cells. Our results indicated that the wild type BMI1 increased the levels of H2A K119Ub and that deletion of the PS region resulted in further increase in H2A K119Ub levels (Fig. 3A). These data suggest that the ∆PS mutant of BMI1 may be physiologically more active. Previously, we reported that the overexpression of BMI1 in normal and immortal cells results in increased proliferation [24,25,34,35]. To determine whether deletion of the PS region further promotes proliferation, we examined the rate of proliferation of MCF10A-B0 control, MCF10A-BMI1WT and MCF10A-BMI1∆PS cells. Our data indeed indicated that MCF10A cells expressing the ΔPS mutant proliferated at a higher rate than the MCF10A-B0 control and MCF10A-BMI1WT cells (Fig. 3B). We also previously reported that BMI1 upregulates AKT in HMECs [45]. Hence, we further examined expression of AKT, GSK3β, cyclin D1 and CDK4 in cells overexpressing the ∆PS mutant of BMI1 and compared it with cells overexpressing the wild type BMI1. The results of western blot analysis suggested that while overexpression of the wild type BMI1 results in modest upregulation of AKT activity (Phospho-AKT) and cyclin D1, the ∆PS mutant further augmented upregulation of AKT and cyclin D1 (Fig. 3C). As compared to overexpression of the wild type (WT) BMI1, no further upregulation of CDK4 was noticed upon overexpression of the ∆PS mutant. As a downstream phosphorylation target of AKT, increased phosphorylation of GSK3β was also evident in cells overexpressing ∆PS mutant of BMI1 (Fig. 3C). No appreciable changes were noticed in the expression of total AKT and total GSK3β (Fig. 3C). Thus, deletion of the PS region of BMI1 further augments the proliferative activity of BMI1.

### Overexpression of the ΔPS mutant of BMI1 results in a partially transformed phenotype in HMECs

Because the ∆PS mutant of BMI1 is more stable and more pro-proliferative, we examined whether its overexpression can transform MCF10A cells. MCF10A cells overexpressing the wild type BMI1 or the ∆PS mutant were seeded in soft agar and examined for colony formation over two weeks. The results indicated that neither overexpression of the wild type BMI1 nor the ∆PS mutant is sufficient to transform MCF10A cells (not shown). Similarly, overexpression of neither the wild type nor the mutant BMI1 was sufficient to form foci in 3T3 fibroblasts (not shown).

The morphology of MCF10A-BMI1∆PS cells appeared to be more fibroblastic than that of the control or the wild type BMI1 overexpressing cells (Additional file 1, Fig. S4). The fibroblastic morphological changes in epithelial cells are suggestive of an EMT phenotype; hence, we analyzed MCF10A-B0, MCF10A-BMI1WT and MCF10A-BMI1∆PS cells for the presence of EMT markers by immunostaining and western blot analysis (Fig. 3D &3E). The results indicated that the MCF10A-B0 control and MCF10A-BMI1 cells expressed E-cadherin, a cell-cell junction protein characteristic of epithelial cells, while MCF10A-BMI1∆PS cells lost the expression of E-cadherin (Fig. 3D &3E). In addition, MCF10A- BMI1∆PS cells also expressed fibroblastic markers such as vimentin and fibronectin (Fig. 3D &3E). These data indicate that the expression of a stable mutant of BMI1 which contains a deletion of the PS domain can induce an EMT phenotype in HMECs. Extensive passaging of the wild type BMI1 expressing cells, particularly at passages more than 15 after drug selection, also exhibited partial EMT and loss of E-cadherin expression (not shown). Since extensive passaging of cells in culture can result in the accumulation of additional stochastic lesions, it is possible that such lesions cooperate with the wild type BMI1 to inhibit E-cadherin expression and induce a partial EMT phenotype.

**Figure 4:**
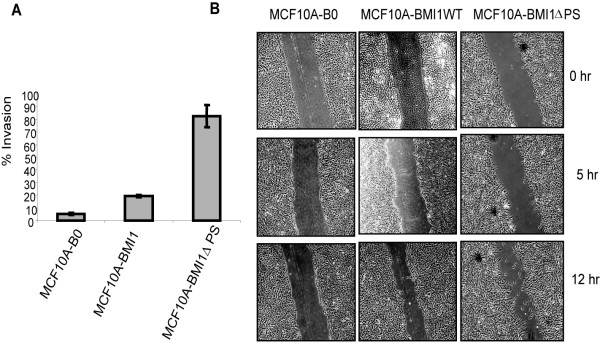
**The ∆PS mutant of BMI1 promotes invasion and migration**. (A) MCF10A-B0, MCF10A-BMI1WT and MCF10A-BMI1∆PS were analyzed for invasion potential using 24-well BD Biocoat Matrigel Invasion Chambers (BD Biosciences, San Jose, CA) as described in the Materials and Methods section. The columns represent mean value of triplicate samples and bars represent SD. (B). The migration potential of MCF10A-derived cells was determined using a wound healing assay as described in the Materials and Methods section.

Next, we performed invasion and wound healing assays to determine the migratory potential of control and MCF10A cells overexpressing either the wild type BMI1 or the ∆PS mutant. The results indicated that MCF10A-BMI1∆PS cells are highly invasive compared to control cells, while MCF10A-BMI1 exhibited a modest invasive potential (Fig. 4A). Similarly, the results of the wound healing assay suggest that the wild type BMI1 expressing cells only exhibit a minimal migration, while MCF10A-BMI1∆PS cells possess the highest migration potential and that these cells filled the wound quickly compared to the MCF10A-B0 control cells (Fig. 4B). Thus, our data suggest that overexpression of the ∆PS mutant of BMI1 leads to acquisition of migration and invasion properties indicative of the partial transformation of HMECs.

**Figure 5:**
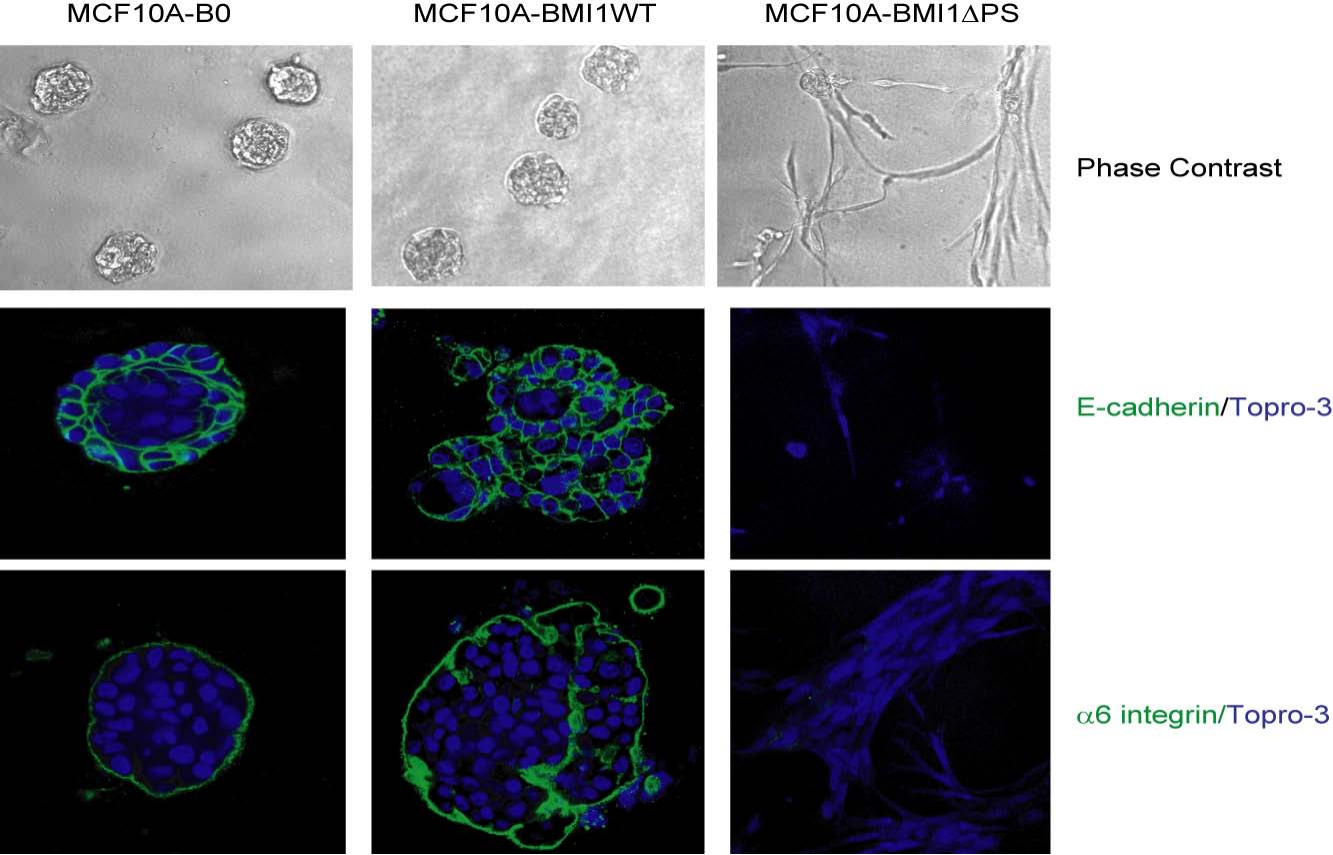
**MCF10A cells overexpressing the ∆PS mutant of BMI1 (MCF10A-BMI1∆PS) exhibit an invasive phenotype in 3-D Matrigel culture**. MCF10A-B0, MCF10A-BMI1WT and MCF10A-BMI1∆PS cells were cultured in chamber slides in 2% Matrigel for 12 days and studied for Matrigel phenotype. Top panel shows the phase contrast images of the acini at day 12. At day 12, the cultures were fixed and immunostained using antibodies specific for E-cadherin (middle panel) and anti-α6 integrin (bottom panel). The nuclei were stained with Topro-3. After staining, acini were photographed (40X) using a Nikon Eclipse 80i confocal microscope.

To further confirm the partial transformation of HMECs by the ∆PS mutant of BMI1, we analyzed cells for 3-D (3-dimensional) growth in Matrigel. Our data indicated that indeed ∆PS mutants expressing MCF10A cells are partially transformed (Fig. 5). Specifically, the MCF10A-B0 control cells formed normal round acini, while the wild type BMI1 expressing MCF10A cells formed slightly irregular acini, and the ΔPS mutant expressing MCF10A cells formed a highly branched structure (Fig. 5A). Next, we performed E-cadherin and α6 integrin immunostaining together with Topro-3 nuclear staining of the acini. The results showed that MCF10A-B0 cells form normal acini with a proper lumen; MCF10-BMI1 cells form irregular acini filled with cells, while there is no lumen-like structure present at all in the ΔPS mutant expressing MCF10A cells (Fig. 5B). Furthermore, the MCF10A-BMI1ΔPS cells were devoid of E-cadherin and α6 integrin confirming induction of EMT and a partially transformed phenotype in these cells (Fig. 5).

### Overexpression of the ∆PS mutant of BMI1 results in bypass of senescence in HDFs

**Figure 6:**
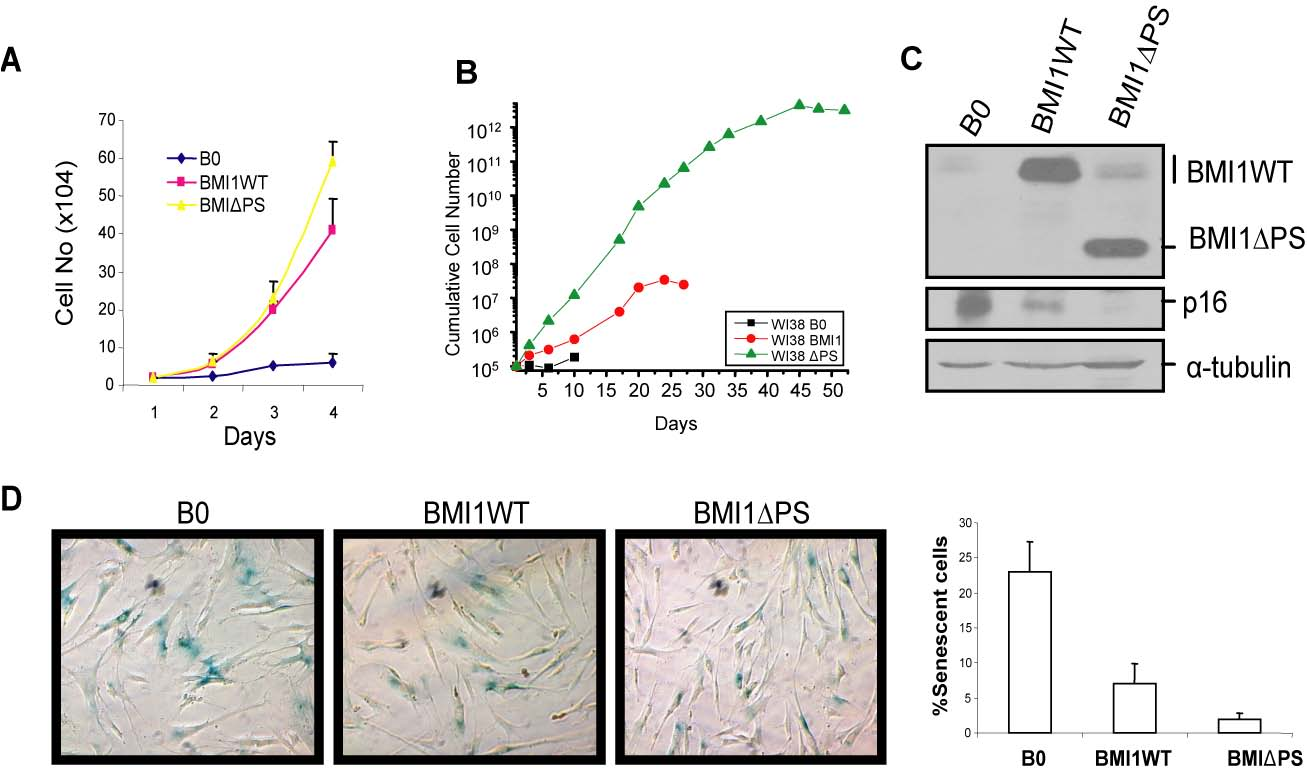
**The ∆PS mutant of BMI1 promotes proliferation and bypasses senescence more efficiently than the wild type BMI1 in HDFs**. (A). WI-38 cells expressing wild type BMI1 or ∆PS mutant of BMI1, and control WI-38-B0 cells were plated at 1 × 10^4 ^cells/well in multi well plates, and harvested and counted after indicated number of days. Proliferation curves were generated by plotting the number of cells against the number of days. (B). Mid passage control B0, and wild type BMI1- and ΔPS mutant-expressing cells were generated and serially passaged in culture to determine the effect of the mutant BMI1 on the long term replicative life span of WI-38 cells. After selection, at each passage 10^5 ^cells were passaged in a T25 and cultures were grown until 70-80% confluence. At each passage, cell numbers were counted and the proliferation curve was generated by plotting cumulative cell number vs days. (C). WI-38-derived cells were analyzed for the expression of p16INK4a, BMI1 (wild type and mutant), and α-tubulin (loading control) by western blot analysis as described in the Materials and Methods section. (D). The number of senescent cells in WI-38-B0, WI-38-BMI1 WT and WI-38-BMI1 ∆PS culture at passage 3 (after selection) was determined using SA-β-gal staining as described in the Materials and Methods section. After staining, the cells were photographed (10X) under phase contrast using a light microscope. The number of senescent cells and total number of cells in each culture were counted in multiple fields to determine the percentage of senescent cells.

Since MCF10A cells are immortal and do not undergo replicative senescence, we used HDFs for examining the effect of overexpression of the ∆PS mutant on senescence. WI-38 cells stably expressing the wild type BMI1 or the PS mutant of BMI1 were generated as described in the Materials and Methods section. After selection and culturing for an additional passage, WI-38-derived cells were analyzed for proliferation and bypass of senescence. Results of proliferation assays carried over 4 days indicated that WI-38 cells expressing ∆PS mutant proliferated at a faster rate compared to the wild type BMI1 expressing cells, which proliferated better than WI-38-B0 control cells (Fig. 6A). We also performed long-term proliferation assays of mid passage WI-38 cells expressing the wild type BMI1 or the ΔPS mutant. The cells were continually passaged in culture and the cumulative cell numbers at each passage were determined to derive a long-term growth/proliferation curve. The results indicated that the WI-38 cells expressing ΔPS mutant proliferated for more than 50 days before undergoing senescence, while the control B0 and wild type BMI1-expressing cells underwent senescence within 15 days and 25 days of growth after selection respectively (Fig. 6B).

Since BMI1 is known to downregulate p16INK4a in HDFs, we analyzed WI-38-B0, WI-38-BMI1WT and WI-38-BMI1PS cells for the expression of p16INK4a. Our data indicated that, as expected, overexpression of the wild type BMI1 led to downregulation of p16INK4a. Importantly, WI-38-BMI1∆PS cells exhibited even further downregulation of p16INK4a (Fig. 6C). Significant downregulation of p16INK4a by the ∆PS mutant was also noticed in hTERT-immortalized WI-38 cells (Additional file 1, Fig. S5). Because p16INK4a regulates senescence and BMI1 downregulates p16INK4a, cells expressing the wild type BMI1 or the ∆PS mutant, and control WI-38-B0 cells were analyzed at the same passage number after puromycin selection for the induction of spontaneous senescence using SA-β-gal marker [46]. Consistent with our earlier data [35], overexpression of BMI1 resulted in significantly fewer senescent cells when compared to control B0 cells (Fig. 6D). Importantly, overexpression of the ∆PS mutant of BMI1 further decreased the number of senescent cells indicating more efficient bypass of senescence in HDFs (Fig. 6B, D). Thus our data indicate that the ∆PS mutant of BMI1 is physiologically more active and pro-proliferative as compared to the wild type BMI1. With respect to proliferation and senescence, similar results were obtained in MRC5, which is another commonly used strain of HDFs (Additional file 1, Fig. S6).

## Discussion

The mouse *Bmi1 *gene was cloned by two different groups in 1991 [5,6], and shortly after that, the human homologue of mouse Bmi1 was cloned [32]. Since then most of the studies on BMI1 have been focused on its role in oncogenesis and stem cell phenotype, and very few studies have analyzed the regulation of BMI1. Only recently, we and others analyzed the transcriptional regulation of *BMI1 *[47,48]. At present, virtually nothing is known about additional transcriptional or posttranslational regulation of BMI1. Such regulation of BMI1 is likely to be highly relevant for the role of BMI1 in development and pathobiology of various diseases, including cancer.

The initial characterization of mouse and human BMI1 predicted the presence of three structural domains in BMI1; a RING finger domain, an HTH domain and a potential PEST domain. Except for the RING finger domain, the functional role of these domains is largely undefined. The RING finger domain is most recognizable domain in BMI1. Although such domains are found in E3 ubiquitin ligases, BMI1 by itself lacks E3 ubiquitin ligase activity. Nonetheless, this domain has been shown to be functionally important [15,34,35,49].

A RING finger domain mutant of Bmi1 (the mouse homologue of human BMI1) was shown to be ineffective in collaborating with c-myc to induce B- and T- cell lymphomas [49]. Moreover, the deletion of the RING finger domain of BMI1 may lead to dominant negative activity of the mutant BMI1 [35], as the overexpression of the ΔRF mutant of BMI1 induces p16INK4a expression and premature senescence, whereas the expression of the wild type BMI1 suppresses p16INK4a expression and bypasses senescence in HDFs [35]. Alkema et al. also showed that in addition to the RING finger domain, a centrally located HTH domain of Bmi1 was also required for B- and T- cell lymphomagenesis by Bmi1 and c-myc [49]. Interestingly, the PS region of Bmi1 was not required to induce B- and T- cell lymphomas in the mouse model [49]. Thus, at present, it is not clear what role the PS domain of BMI1 plays in oncogenesis.

In the present study, we report that deletion of the PS domain of BMI1 increases its half life and that the mutant protein becomes very stable. Although naturally occurring deletions in the PS region of BMI1 have not been reported, our studies form the basis of detailed analysis of posttranslational regulation of BMI1 and biochemical analysis of its degradation pathways. With respect to biochemical analysis of BMI1 proteolysis, we noticed the presence of a degron motif DSG(X)_2+n_S, in the PS domain of BMI1. The degron motif is a recognition site for the βTRCP (β-transducin repeat containing protein), which is a member of the F-box protein family and constitutes one of the subunit of the ubiquitin ligase complex known as SCF (SKP-Cullin-F-Box) [50]. The SCF complex is involved in proteosome-mediated degradation of several proteins known to be involved in cell cycle and oncogenesis such as CDC25A [51-53], Gli [54,55] Mcl-1 [56], and PDCD4 [57]. It is tempting to speculate that a βTRCP-based SCF complex posttranslationally regulates the stability of BMI1. Identification and detailed characterization of such an SCF complex and its constituents remain to be undertaken.

We reasoned that if the deletion of the PS region makes BMI1 more stable, overexpression of the ∆PS mutant of BMI1 may augment known biological activities of the wild type protein and/or exhibit additional biological activities. The best known biological activity of BMI1 is regulation of proliferation, which it upregulates via p16INK4a-dependent and -independent pathways. The p16INK4a-independent pathway includes AKT and its targets, and possibly other growth regulatory genes. Indeed, our results indicate that deletion of the PS domain of BMI1 results in augmentation of pro-proliferative activity of BMI1 via both p16INK4a-dependent and -independent pathways in different cell types. Specifically, overexpression of the ∆PS mutant in HMECs results in upregulation of AKT and its targets such as cyclin D1, and increased proliferation. On the other hand in primary HDFs, which express p16INK4a, overexpression of the ∆PS mutant results in significant downregulation of p16INK4a accompanied by increase in proliferation and decrease in senescence.

Apart from the increase in proliferation in both epithelial cells and fibroblasts, overexpression of the ∆PS mutant also results in induction of an EMT phenotype, which is often accompanied by downregulation of E-cadherin and upregulation of fibroblastic markers such as fibronectin and vimentin. The EMT phenotype is thought to be important not only for development of an organism but also for certain pathological conditions such as cancer and organ fibrosis [58]. PcG proteins, in particular EZH2 has been shown to downregulate E-cadherin in breast and prostate cancer cells [59,60]. In addition, a recent report suggests that BMI1 can downregulate E-cadherin expression and induce EMT via downregulation of PTEN in nasopharyngeal epithelial cells (NEPC) [61]. However, our unpublished data suggests that in HMECs, overexpression of the wild type BMI1 is not sufficient to downregulate PTEN. It is possible that unidentified potential pro-oncogenic lesions in immortalized NEPC and nasopharengeal carcinoma cooperate with the wild type BMI1 to downregulate PTEN expression and induce EMT. A different recent report suggests that BMI1 interacts with nuclear PTEN [62]. Interaction of BMI1 with PTEN may also inhibit its function, and this interaction in part could induce an EMT phenotype. Stable BMI1 in the form of the ∆PS mutant may also upregulate EZH2, which in turn could directly downregulate the *CDH1 *promoter and induce EMT. These hypotheses remain to be examined. In any case, it appears that by increasing BMI1 stability via deletion of its negative regulatory sequences, one can potentially induce EMT; although the mechanism of this induction is not clear at this point.

Although the exact role of the PS domain of BMI1 in the pathobiology of cancer remains to be explored, our studies imply that under certain pathological conditions, BMI1 may be more stable due to deregulation of its degradation machinery, which is likely to be regulated by the sequences present in the PS region of BMI1. So far there is no report of somatic oncogenic mutations or deletions occurring in the PS region of BMI1; however such a possibility cannot be ruled out. In summary, our data implicates the PS domain of BMI1 in its negative regulation, possibly via posttranslational mechanisms. Based on our new and published data, we propose that the RING finger domain of BMI1 can be classified as a catalytic domain, while the PS domain of BMI1 can be termed as a regulatory domain. In view of the well established roles of BMI1 in oncogenesis and stem cell maintenance, our studies have important implications for the pathobiology of cancer and the development of novel treatment strategies for cancer patients.

BMI1 and its target p16INK4a are also important regulators of in vivo aging. Recently it was shown that *Bmi1-/- *mice present several growth defects, progeroid phenotype, such as hair loss, lens cataracts and reduced locomotor activity, and a high lethality rate exceeding 75% [63]. Furthermore, the levels of p16INK4a , which is a well established target of BMI1, but not other CDK inhibitors increase with aging in vivo, and inhibition of p16INK4a can ameliorate the physiological impact of aging on stem cells and improve injury repair in aged tissues [64-68]. As the deletion of the PS domain of BMI1 results in a stable and constitutively active BMI1, and leads to a robust downregulation of p16INK4a, we speculate that the PS region of BMI1 could also be targeted for the development of treatment strategies for age-related pathologies.

## Methods

### Cells, cell culture methods, and senescence and proliferation assays

The MCF10A cell line was obtained from ATCC (Manassas, VA, USA), and cultured as described previously [69]. WI-38 and MRC5 strains of HDFs were obtained from National Institute on Aging (NIA), Aging Cell Repository, Coriell Institute for Medical Research, Camden, NJ. HDFs were passaged in culture in DMEM containing 10% FCS, 2 mM L-glutamine at 37°C under 10% CO_2 _and 90% atmosphere as described [35,46,47,70]. The induction or bypass of senescence in HDFs expressing wild type or mutant BMI1 proteins was determined using histochemical staining for senescence-associated beta galactosidase (SA-β-gal) marker as described [35,46,47,70]. Similarly, short and long term proliferation assays were done as described [35,46,47,70].

### Retrovirus production, infection and generation of cells expressing wild type or mutant BMI1 proteins

The cDNA of mutant BMI1 protein lacking the PS domain was generated by PCR and cloned into pBabe-puro retroviral vector to generate pBabe-BMI1ΔPS. The pBabe-BMI1 WT (wild type), pBabe-BMI1 ΔRF (RING finger deleted) and pBabe-BMI1ΔHT (HTH domain deleted) have been described previously [34,35] (Fig. 1A). Retroviruses expressing wild type or mutant BMI1 proteins were produced by transiently transfecting retroviral vectors together with a packaging plasmid pIK into a packaging cell line tsa54 as described [70]. After 24 hrs of transfection, retrovirus-containing supernatant was collected, filtered, and directly used to infect recipient cells or was stored at -80°C as described [70]. After three sequential infections, recipient cells were passaged in medium containing puromycin (0.5-1 μg/ml) as described [35,46,47,70]. The pool populations of drug-selected cells were further passaged in culture and analyzed as necessary.

### Antibodies and western blot analyses, and protein half life determination

Most of the antibodies were obtained from commercial sources. These included; anti-BMI1 mAb (monoclonal antibody) 1H6B10G7 from Invitrogen Corporation (Camarillo, CA), anti-E-cadherin mAb and anti-vimentin mAb from BD Biosciences (San Jose, CA), anti-α6 integrin mAb from Chemicon Corporation (now Millipore, Billerica, MA), and mAb against β-actin from Sigma-Aldrich (St. Louis, MO). Additionally, pAbs (polyconal antibodies) against CDK4, phospho-AKT 1/2/3 (Ser-473), and AKT-1/2/3, and mAbs against α-tubulin, E-cadherin, fibronectin, cyclin D1 and p16INK4a were obtained from Santa Cruz Biotechnology (Santa Cruz, CA). Antibodies against Phospho-GSK3β (Ser9) and total GSK3β were obtained from Cell Signaling Technologies (Danvers, MA).

Western blot analyses of total cell extracts from control and wild type or mutant BMI1- expressing cells using various antibodies were performed as described [47]. The half life of wild type and mutant BMI1 protein was determined using cyclohexamide (CHX) treatment of MCF10A cells expressing BMI1 proteins as described [47]. Briefly, sub confluent MCF10A cells expressing wild type or mutant BMI1 proteins were treated with 100 μg/mL CHX for various time points (0-240 min) to stop the synthesis of new proteins. After the CHX-treatment, cell lysates were prepared and analyzed for the expression of BMI1 and α-tubulin. The normalized % residual BMI1 was plotted against the different time points to determine the rate of proteolysis and the half-life of BMI1. For the analysis of H2A K119Ub, total histones were prepared using acid extraction method as described [71], and western blot analysis was carried out using a pAb against H2A K119Ub (Millipore Inc., Billerica, MA).

### Soft agar, invasion, migration and wound healing assays

Anchorage-independent growth assay using soft agar, and invasion and migration assays were performed as described [24,69]. Briefly, for invasion assays, 2.5 × 10^4 ^cells in 0.2 ml medium were added to the top chambers of Matrigel-coated or control wells (BD Biosciences, San Jose, CA), with D-medium containing EGF in the bottom chamber. After 24 hr at 37°C, the cells on the top side were removed by scraping, and the invaded cells were fixed in methanol at -20°C and visualized using the Diff-Quik stain (Dade Behring, Deerfield, IL). Live images were taken under 10X magnification for counting of cells that invaded through the Matrigel. The experiments were done in triplicates and data were analyzed using the Student's *t *test, for which P < 0.05 was considered significant. For the wound healing assay, the MCF10A-derived cells were grown to 90% confluence in growth factor deprived D3 medium [24,69]. A wound was made in the middle of culture dish containing near-confluent cells and the cells were stimulated with EGF containing D medium. Cells were photographed at 0 hr, before adding D medium and at 5 and 12 hr, after stimulating with D medium. Cells were photographed using a light microscope (4X).

### Immunofluorescence and Matrigel assays

Immunofluorescence (IF) and Matrigel assays were performed as described [24,69]. Briefly, cells were plated in chamber slides. Cells were fixed in 4% formaldehyde, permeabilized with 0.5% Triton X-100 for 5 min, and immunostained with anti-E-cadherin, anti-fibronectin, and anti-vimentin antibodies followed by staining with Alexa Fluor 488- and Alexa Fluor 594-conjugated secondary antibodies (Molecular Probes Inc. Eugene, OR) as required. The slides were mounted with Vectashield mounting medium containing 4',6-diamidino-2-phenylindole (DAPI) (Vector Laboratories). After staining, the cells were photographed (40X) using a Nikon Eclipse 80i confocal microscope. To perform Matrigel assays, cells were plated in 2% Matrigel in a chamber slide on top of a polymerized layer of 100% Matrigel. The cultures were fed every 3 days. Phase-contrast images were obtained under 10X magnification. For immunofluorescence analyses, the cultures were fixed in 4% paraformaldehyde on day 12, permeabilized with 0.5% Triton X-100 for 5 min, and stained with anti-E-cadherin or anti-α6 integrin primary antibodies followed by Alexa Fluor 488-conjugated secondary antibodies (Molecular Probes). Nuclei were stained with Topro-3 and slides were mounted with Vectashield mounting medium (Vector Laboratories). Images were acquired with a Nikon Eclipse 80i confocal microscope.

## Competing interests

The authors declare that they have no competing interests.

## Authors' contributions

GPD conceived, coordinated the study, interpreted data and wrote the manuscript. AKY, AAS, MD, PVB and RS performed all the experiments, and helped in interpretation of the data and the preparation of the manuscript. All authors read and approved the final manuscript.

## Supplementary Material

Additional file 1 Supplementary data. The additional file contains Figure legends and Figures S1-S6. Click here for file
