# Supplementary material for: Deletion analysis of BMI1 oncoprotein identifies its negative regulatory domain
Source: Mol Cancer. 2010 Jun 22;9:158. doi: 10.1186/1476-4598-9-158 (PMC2900245; doi:10.1186/1476-4598-9-158)

Supplementary Figures

Figure S1: (A). Total cell lysate of the wild type BMI1 overexpressing cells was left untreated or treated with Calf Intestinal Phosphatase (NEB, 100 Units) for 1hr, and analyzed for the expression pattern of BMI1 by western blot analysis. (B). MCF10A-BMI1WT cells were treated with PMSF (2 mM), Aprotinin (2 μg/ml) and Lactacystin (5 μM) for 1 hr to determine their effect on wild type BMI1. Cell lysates were analyzed for the accumulation of BMI1 by western blot analysis. The accumulated proteins were quantified by densitometric analysis of signal present in respective lanes and by normalizing it to the individual α-tubulin signals. Densitometric analysis was done as described in Figure1B (main text).

Figure S2 and S3: The ΔPS mutant is highly stable in MRC5 (S1) and IMR90 (S2) strains of HDFs. HDFs expressing endogenous wild type BMI1 (B0), overexpressed wild type BMI1 (BMI1WT) and the ΔPS mutant of BMI1 (BMI1ΔPS) were treated with CHX for the indicated time points (min), and the half life of wild type or mutant BMI1 protein was determined as described in the Materials and Methods section of the main text.

Figure S4: The morphology of MCF10A-derived cells (MCF10A-B0 (control), MCF10A-BMI1 and MCF10A-BMI1ΔPS was examined by phase contrast microscopy. In each case, freshly plated cells after 24 hr in culture were photographed (4X).

Figure S5: The increased downregulation of p16INK4a by the ΔPS mutant of BMI1 as compared to wild type BMI1 was confirmed in WI-38hTERT (telomerase immortalized WI-38). Expression of BMI1, p16INK4a and α-tubulin was determined by western blot analysis as described in the Materials and Methods section of the main text.

Figure S6: As compared to wild type BMI1, the ΔPS mutant of BMI1 further increased short term and long term proliferation and decreased the number of spontaneously arising senescent cells in MRC5 fibroblasts. Proliferation and senescence assays were done as described in the Materials and Methods section of the main text and figure legend of the Fig. 6.

Suppl. Fig. S1

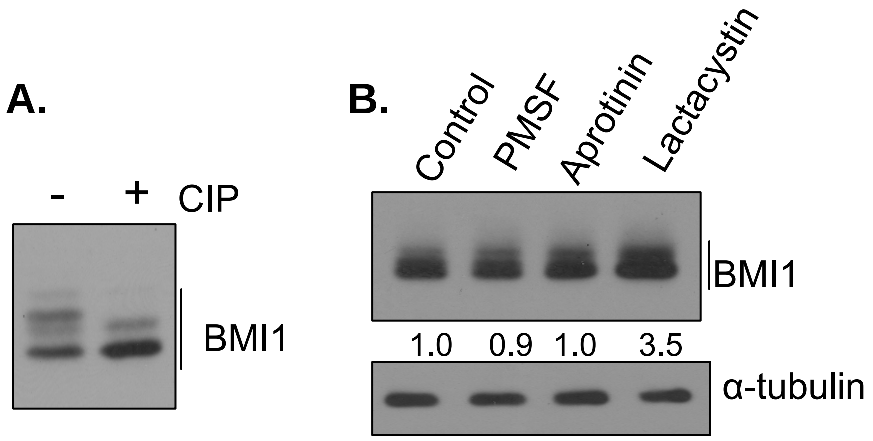
A.
-
+
CIP
BMI1
B.
Control
PMSF
Aprotinin
Lactacystin
BMI1
1.0
0.9
1.0
3.5
α-tubulin

Suppl. Fig. S2

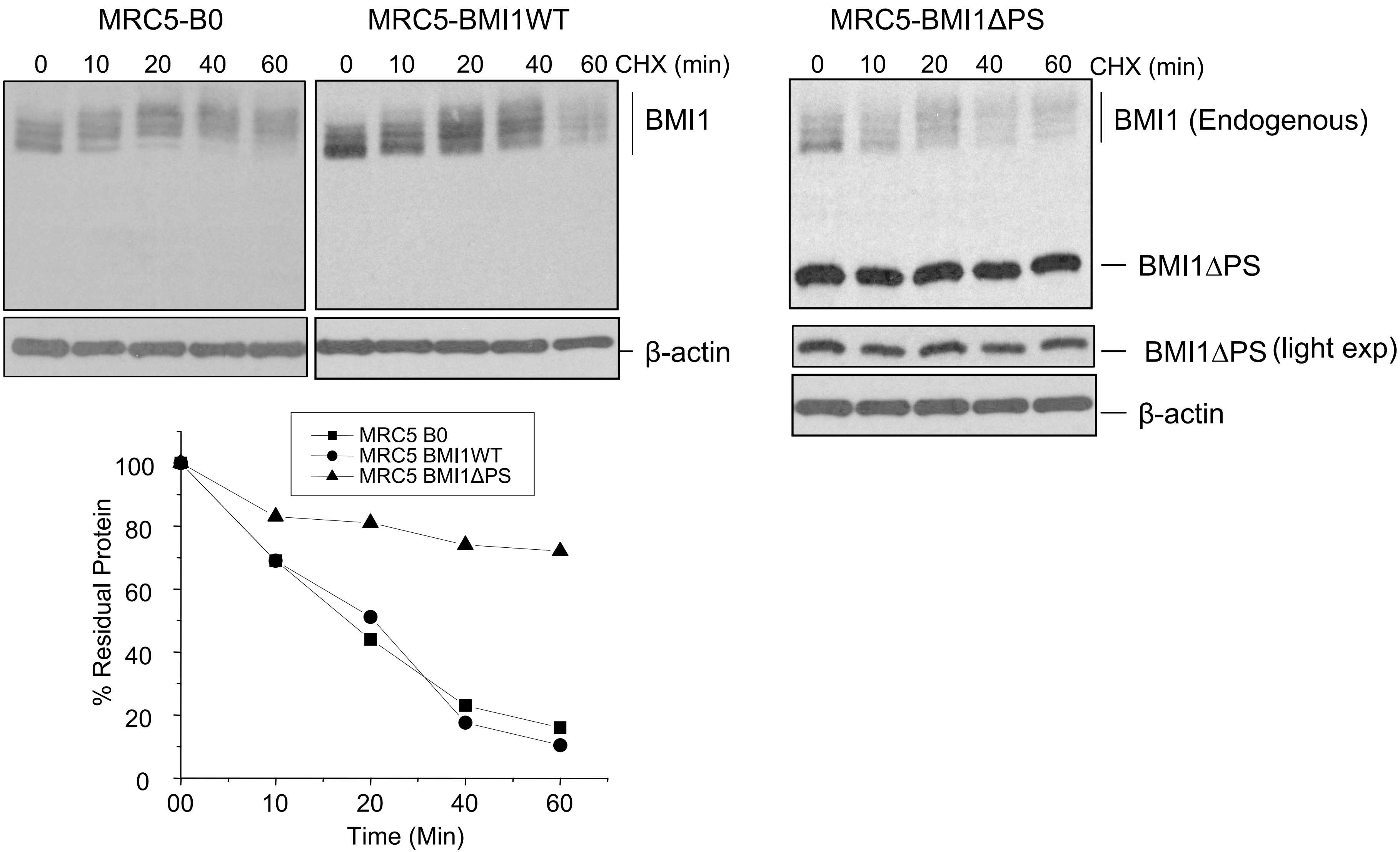
MRC5-B0
MRC5-BMI1WT
MRC5-BMI1ΔPS
0 10 20 40 60 0 10 20 40 60 CHX (min)
0 10 20 40 60 CHX (min)
BMI1
BMI1 (Endogenous)
BMI1ΔPS
BMI1ΔPS (light exp)
β-actin
β-actin
MRC5 B0
MRC5 BMI1WT
MRC5 BMI1ΔPS
% Residual Protein
0
20
40
60
80
100
00 10 20 40 60
Time (Min)

Suppl. Fig. S3

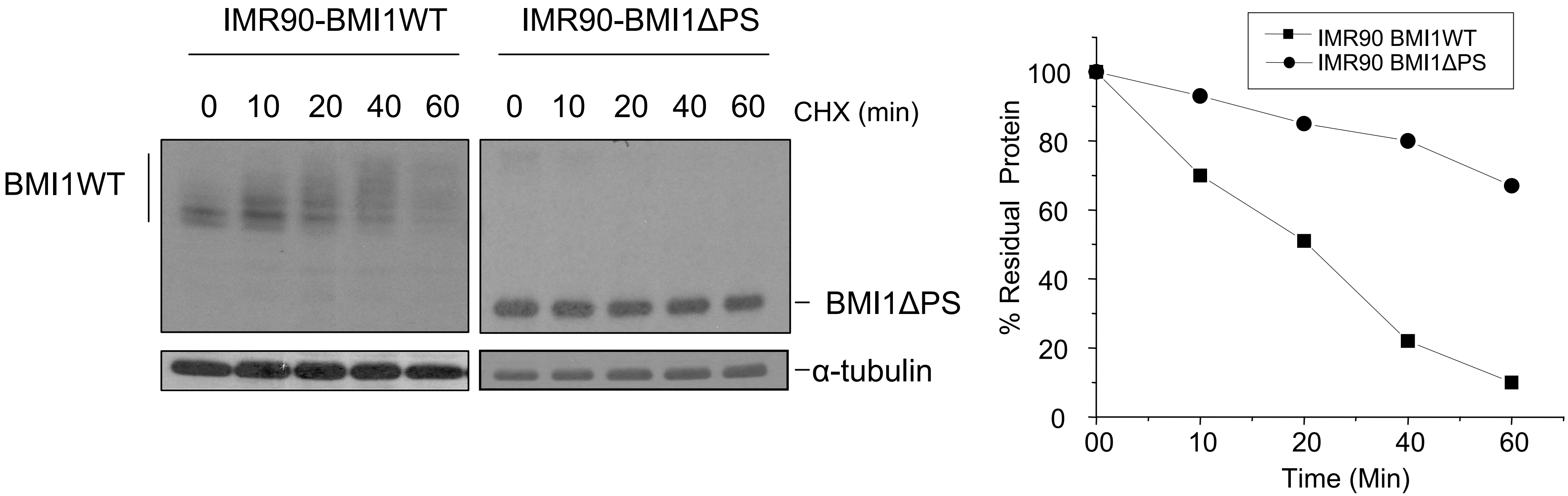
IMR90-BMI1WT
IMR90-BMI1ΔPS
0 10 20 40 60 0 10 20 40 60 CHX (min)
BMI1WT
BMI1ΔPS
α-tubulin
IMR90 BMI1WT
IMR90 BMI1ΔPS
% Residual Protein
100
80
60
40
20
0
00 10 20 40 60
Time (Min)

Suppl. Fig. S4

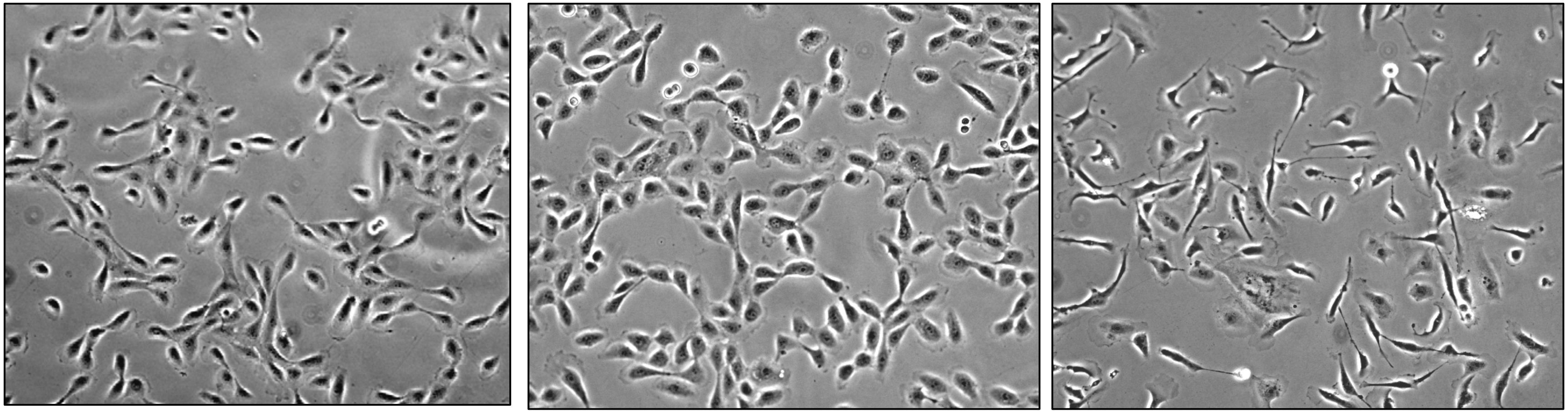

MCF10A-B0 MCF10A-BMI1 MCF10A-BMI1ΔPS

Suppl. Fig. S5

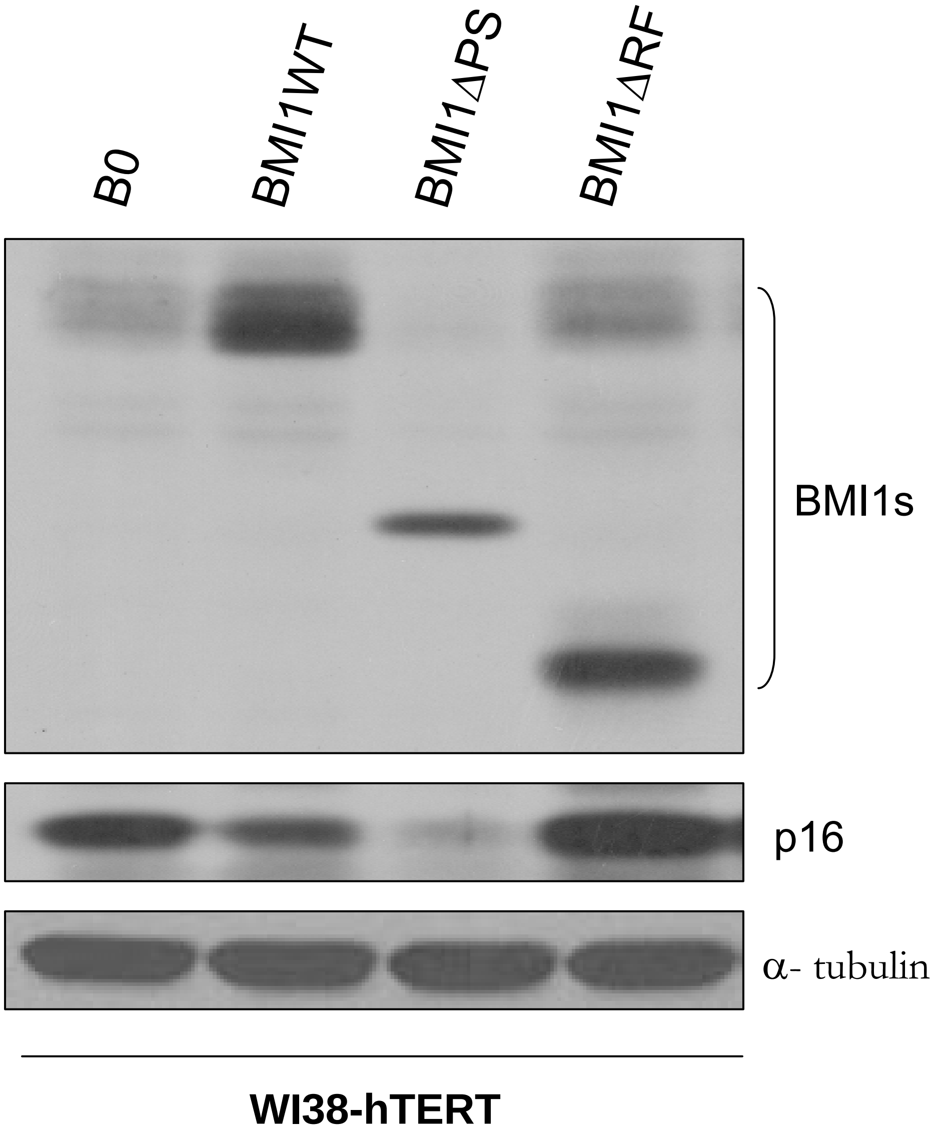

Suppl. Fig. S6

A.

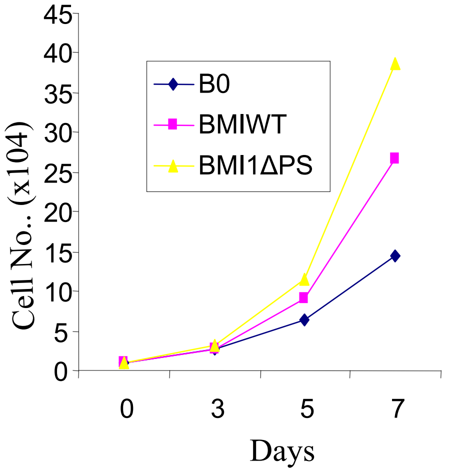

B.

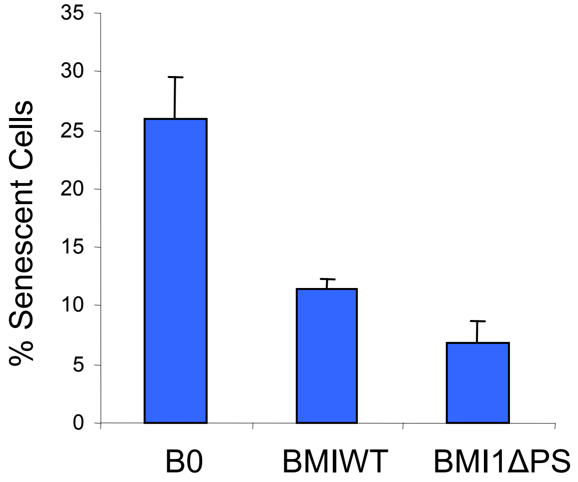

C.

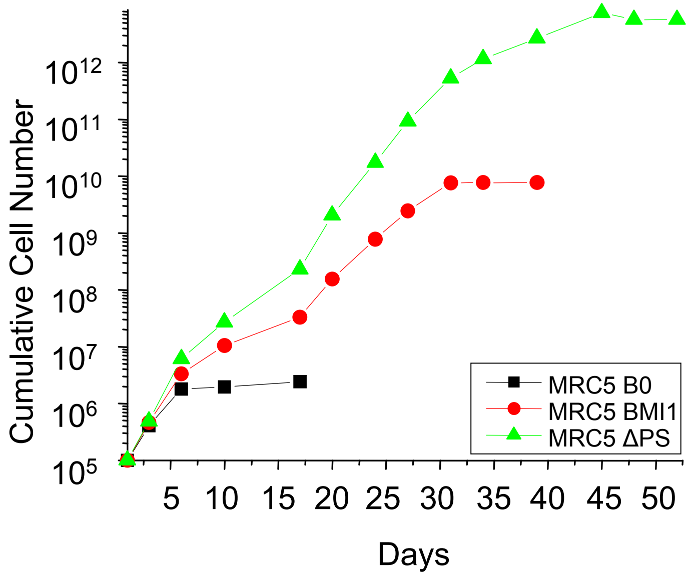

Supplement: Additional file 1 — Supplementary data. The additional file contains Figure legends and Figures S1-S6. [file 1476-4598-9-158-S1.PDF]
